# The shunt from the cyclooxygenase to lipoxygenase pathway in human osteoarthritic subchondral osteoblasts is linked with a variable expression of the 5-lipoxygenase-activating protein

**DOI:** 10.1186/ar2092

**Published:** 2006-12-08

**Authors:** Kelitha Maxis, Aline Delalandre, Johanne Martel-Pelletier, Jean-Pierre Pelletier, Nicolas Duval, Daniel Lajeunesse

**Affiliations:** 1 Unité de recherche en Arthrose, Centre de recherche du Centre Hospitalier de l'Université de Montréal, Hôpital Notre-Dame, 1560 rue Sherbrooke Est, Montréal, Québec, Canada, H2L 4M1

## Abstract

Osteoarthritis (OA) is characterized by articular cartilage degradation and hypertrophic bone changes with osteophyte formation and abnormal bone remodeling. Two groups of OA patients were identified via the production of variable and opposite levels of prostaglandin E_2 _(PGE_2_) or leukotriene B_4 _(LTB_4_) by subchondral osteoblasts, PGE_2 _levels discriminating between low and high subgroups. We studied whether the expression of 5-lipoxygenase (5-LO) or 5-LO-activating protein (FLAP) is responsible for the shunt from prostaglandins to leukotrienes. FLAP mRNA levels varied in low and high OA groups compared with normal, whereas mRNA levels of 5-LO were similar in all osteoblasts. Selective inhibition of cyclooxygenase-2 (COX-2) with NS-398-stimulated FLAP expression in the high OA osteoblasts subgroup, whereas it was without effect in the low OA osteoblasts subgroup. The addition of PGE_2 _to the low OA osteoblasts subgroup decreased FLAP expression but failed to affect it in the high OA osteoblasts subgroup. LTB_4 _levels in OA osteoblasts were stimulated about twofold by 1,25-dihydroxyvitamin D_3 _(1,25(OH)_2_D_3_) plus transforming growth factor-β (TGF-β), a situation corresponding to their effect on FLAP mRNA levels. Treatments with 1,25(OH)_2_D_3 _and TGF-β also modulated PGE_2 _production. TGF-β stimulated PGE_2 _production in both OA osteoblast groups, whereas 1,25(OH)_2_D_3 _alone had a limited effect but decreased the effect of TGF-β in the low OA osteoblasts subgroup. This modulation of PGE_2 _production was mirrored by the synthesis of COX-2. IL-18 levels were only slightly increased in a subgroup of OA osteoblasts compared with normal; however, no relationship was observed overall between IL-18 and PGE_2 _levels in normal and OA osteoblasts. These results suggest that the shunt from the production of PGE_2 _to LTB_4 _is through regulation of the expression of FLAP, not 5-LO, in OA osteoblasts. The expression of FLAP in OA osteoblasts is also modulated differently by 1,25(OH)_2_D_3 _and TGF-β depending on their endogenous low and high PGE_2 _levels.

## Introduction

Osteoarthritis (OA) is the leading cause of disability among the elderly population [1]. Despite its prevalence, we still do not fully understand the etiology, pathogenesis and progression of this disease [2,3]. OA progresses slowly and has a multifactorial origin. The disease is characterized by the degradation and loss of articular cartilage, and hypertrophic bone changes with osteophyte formation and subchondral plate thickening [4,5]. It includes changes in articular cartilage and surrounding bone, and an imbalance in loss of cartilage through matrix degradation and an attempt to repair this matrix [4,5]. Specific interactions between bone and cartilage have not been clearly defined in OA; however, there is mounting evidence to indicate a direct intervention of the bone compartment in the initiation and progression of OA [6-8]. We already identified that a growth factor, hepatocyte growth factor, is produced more abundantly by OA osteoblasts than by normal osteoblasts, yet hepatocyte growth factor accumulates in cartilage matrix and is more abundant in OA cartilage [9].

As the initiating events leading to OA are still poorly defined, clinical intervention still targets the reduction of pain and discomfort in afflicted patients. Conventional non-steroidal anti-inflammatory drugs (NSAIDs) inhibit cyclooxygenases (COX-1 and/or COX-2), the key enzymes that metabolize arachidonic acid into prostaglandins and thromboxanes [10,11]. The decrease in prostaglandin and thromboxane levels is probably the basis for the anti-inflammatory and analgesic activity of the NSAIDs that are widely used for the treatment of OA. Newer drugs (coxibs) have in recent years targeted the selective reduction of COX-2 activity because this inducible form is expressed in response to inflammation, and coxibs are safer for the gastrointestinal tract. However, long-term inhibition of COX-2 could lead to a shunt to the 5-lipoxygenase (5-LO) pathway, as we observed *in vitro *with OA osteoblasts [12], leading to the formation of leukotrienes (LTs), which can induce gastric lesions and ulceration [13,14]. The local production of LTs in joint tissues is detrimental to tissues such as the subchondral bone compartment. Indeed, the production of LTs can promote bone resorption [15], and LTs are potent chemoattractants and stimulators of inflammation [16-18]. Moreover, because the safety of coxibs is now in question, this potential long-term effect on LT production could also be detrimental.

Osteoblasts produce prostaglandins by both COX-1 and COX-2 activities [19,20] and also produce LTs [12]. However, the actual levels of prostaglandin E_2 _(PGE_2_) and LTs produced *in vivo *in OA bone tissue are controversial [21,22]. We previously reported that the levels of LTs produced *in vitro *by OA osteoblasts are either similar to or higher than normal as a result of the endogenous production of PGE_2 _by these cells [12], and indeed the endogenous production of PGE_2 _and of IL-6 by OA osteoblasts separated OA patients into two subgroups: those producing normal PGE_2 _levels and those producing high PGE_2 _levels [23]. Moreover, chronic inhibition of COX-2 with a selective inhibitor such as NS-398 in OA osteoblasts enhanced the production of leukotriene B_4 _(LTB_4_) [12], a situation also observed in other cell systems using selective COX-2 inhibitors [24]. Hence, chronic inhibition of COX-2 may promote abnormal 5-LO activity. The exact mechanism involved in this shunt toward the 5-LO pathway remains obscure. The production of LTs requires active 5-LO in the presence of calcium [25,26], yet arachidonic acid must be presented by the 5-LO-activating protein [25,27]. In macrophages, the shunt from the COX to the 5-LO pathway is due to an increase in 5-LO expression [28,29], whereas in alveolar macrophages and in neutrophils it is due to altered 5-lipoxygenase-activating protein (FLAP) expression [30,31]. Whether PGE_2 _directly modulates the production of LTs in osteoblasts remains unknown. However, the inhibition of the production of LTs in macrophages is due to high PGE_2 _levels as a result of an increase in IL-10 [24], whereas in neutrophils IL-18 stimulates the production of LTs [32].

The aim of this study was to explore the mechanisms responsible for the shunt from the COX to the 5-LO pathway in human OA osteoblasts. We also examined the implication of both 5-LO and FLAP in the production of LTB_4 _in these cells and the factors that might modulate their expression. We also sought to determine whether there was a relationship between PGE_2 _levels and either IL-10 or IL-18 levels in OA osteoblasts.

## Materials and methods

### Patients and clinical parameters

Tibial plateaux were dissected away from the remaining cartilage and trabecular bone under sterile conditions from OA patients who had undergone total knee replacement surgery as described previously [23,33-35]. A total of 35 patients (aged 68.8 ± 7.6 years (mean ± SD); 7 males, 28 females) classified as having OA, as defined in the recognized clinical criteria of the American College of Rheumatology, were included in this study [36]. None of the patients had received medication that would interfere with bone metabolism, including corticosteroids, for six months before surgery. A total of 18 subchondral bone specimens of tibial plateaux from normal individuals (aged 62.2 ± 14.3 years (mean ± SD); 11 males, 7 females) were collected at autopsy within 16 hours of death. These were used after it had been established that they had not been on any medication that could interfere with bone metabolism and had not had any bone metabolic disease. Individuals showing abnormal cartilage macroscopic changes and/or subchondral bone plate sclerosis were not included in the normal group. All human materials were acquired after obtaining signed agreement from patients undergoing knee surgery, or from their relatives for the specimens collected at autopsy in accordance with the guidelines of the Clinical Research Ethics Committee of the Centre Hospitalier de l'Université de Montréal.

### Preparation of primary subchondral bone cell culture

Isolation of subchondral bone plate and the cell cultures from the non-sclerotic and sclerotic areas were prepared as described previously [33,34,37,38]. At confluence, cells were passaged once at 25,000 cells/cm^2 ^and grown for 5 days in Ham's F12/DMEM medium (Sigma-Aldrich, Oakville, Ontario, Canada) containing 10% fetal bovine serum before specific assays. Conditioning was performed for a further 24 hours in serum-free Ham's F12/DMEM medium. Confluent cells were incubated in the presence or absence of 1,25-dihydroxyvitamin D_3 _(1,25(OH)_2_D_3_; 50 nM) for 48 hours. Supernatants were collected at the end of the incubation and kept at -80°C before assays. Cells were prepared for either SDS-PAGE separation or RT-PCR experiments. Cells prepared for SDS-PAGE separation were lysed with RIPA buffer (50 mM Tris-HCl pH 7.4, 1% Nonidet P40, 0.5% sodium deoxycholate, 0.1% SDS, 150 mM NaCl containing the following inhibitors: 10 μg/ml aprotinin, 10 μg/ml leupeptin, 10 μg/ml pepstatin, 10 μg/ml *O*-phenanthroline, 1 mM sodium orthovanadate and 1 mM dithiothreitol), and kept at -80°C before assays. Protein determination was performed by the bicinchoninic acid method [39].

### Measurement of PGE_2_, IL-10 and IL-18 content in culture medium

The concentration of PGE_2_, IL-10 and IL-18 was determined in culture medium of confluent cells incubated for their last 48 hours in Ham's F12/DMEM containing 1% ITS. Specific ELISAs from Cayman Chemicals (Ann Arbor, MI, USA) for PGE_2 _(specificity 100%, sensitivity 7.8 pg/ml), from R&D Systems (Minneapolis, MN, USA) for IL-10 and from Medical and Biological Laboratories Co (Nagoya, Japan) for IL-18 (specificity 100% for both, sensitivity 0.5 pg/ml for IL-10 and 12.5 pg/ml for IL-18) were used as described in the manufacturer's manual. All determinations were performed in triplicate for each cell culture.

### RNA extraction and RT-PCR assays

Total cellular RNA from normal and OA osteoblasts was extracted with TRIzol™ reagent (Invitrogen, Burlington, Ontario, Canada) in accordance with the manufacturer's specifications and than treated with the DNA-free™ DNase Treatment and Removal kit (Ambion, Austin, TX, USA) to ensure the complete removal of chromosomal DNA. The RNA was quantified with the RiboGreen RNA quantification kit (Molecular Probes, Eugene, OR, USA). The RT reactions were primed with random hexamers with 2 μg of total RNA in a 100 μl final reaction volume followed by PCR amplification as described previously [35]. 5-LO and FLAP PCR products of 467 and 399 base pairs, respectively, were generated by PCR amplification with the use of 20 pmol of each primer 5'-CTG TTC CTG GGC ATG TAC CC-3' (sense) and 5'-GAC ATC TAT CAG TGG TCG TG-3' (antisense), and 5'-AAT GGG AGG AGC TTC CAG AG-3' (sense) and 5'-ACC AAC CCC ATA TTC AGC AG-3' (antisense). To ensure equivalent loading, glyceraldehyde-3-phosphate dehydrogenase (GAPDH) was amplified in the same solution, with the use of 20 pmol of each primer 5'-CAG AAC ATC ATC CCT GCC TCT-3' (sense) and 5'-GCT TGA CAA AGT GGT CGT TGAG-3' (antisense) to generate a predicted amplified sequence of 360 base pairs [40]. However, the amplification of 5-LO and FLAP mRNA species was performed separately from that of GAPDH mRNA to avoid substrate depletion. Our preliminary results indicated that we were still in the linear portion of the amplification of GAPDH with 25 cycles and of 5-LO or FLAP with 35 cycles; PCR amplifications were therefore performed with these respective numbers of cycles. After amplification, DNA was analyzed on an agarose gel and revealed by ultraviolet detection. Densitometric analysis was performed for each amplimer against that for GAPDH with a ChemiImager 4000 (Alpha Innotech Corporation, San Leandro, CA, USA). The results are presented as the relative expression of Coll1A1 and Coll1A2 normalized to the housekeeping gene GAPDH.

### Real-time PCR

Real-time quantification of 5-LO, FLAP and GAPDH mRNA was performed in the GeneAmp 5700 Sequence Detection System (Applied Biosystems, Foster City, CA, USA) with the 2X Quantitect SYBR Green PCR Master Mix (Qiagen) used in accordance with the manufacturer's specifications. In brief, 100 ng of the cDNA obtained from the RT reactions were amplified in a total volume of 50 μl consisting of 1 × Master mix, 0.5 unit of uracil-*N*-glycosylase (UNG; Epicentre Technologies, Madison, WI, USA) and the gene-specific primers described above, which were added at a final concentration of 200 nM. The tubes were first incubated for 2 minutes at 50°C (UNG reaction), then at 95°C for 15 minutes (UNG inactivation and polymerase activation) followed by 40 cycles each consisting of denaturation (94°C for 15 seconds), annealing (60°C for 30 seconds), extension (72°C for 30 seconds) and data acquisition (77°C for 15 seconds). The data were collected and processed with the GeneAmp 5700 SDS software and given as threshold cycle (*Ct*), corresponding to the PCR cycle at which an increase in reporter fluorescence above baseline signal could first be detected. When comparing normal and OA basal expression levels, the *Ct *values were converted to numbers of molecules and the values for each sample were calculated as the ratio of the number of molecules of the target gene to the number of molecules of GAPDH.

### Western blot analysis of cyclooxygenase-2 (COX-2) levels in OA osteoblasts

Cell extracts were loaded on a 10% polyacrylamide gel and separated by SDS-PAGE under reducing conditions [41]. The proteins were then transferred electrophoretically to a poly(vinylidene difluoride) membrane (Boehringer Mannheim, Penzberg, Germany), and Western blotting was performed as described in the Enhanced Chemiluminescence (ECL) Plus detection system's manual (Pierce, Brockville, Ontario, Canada). COX-2 levels were determined with a polyclonal rabbit anti-human COX-2 antibody (Cayman Chemical-Cedarlane, Hornby, Ontario, Canada) at a 1:400 dilution. The secondary antibody used for the detection of the rabbit anti-human COX-2 was a goat anti-rabbit IgG (1:20,000 dilution) from Upstate Biotechnology (Lake Placid, NY, USA). Densitometric analysis of Western blot films was performed with a Macintosh MacOS 9.1 computer using the public-domain NIH Image program developed at the US National Institutes of Health with the Scion Image 1.63 program [42].

### Phenotypic characterization of human subchondral osteoblast cell cultures

Phenotypic features of osteoblast cultures were determined by evaluating 1,25(OH)_2_D_3_-dependent (50 nM) alkaline phosphatase activity and osteocalcin release. Alkaline phosphatase activity was determined on cell aliquots by substrate hydrolysis with *p*-nitrophenyl phosphate, and osteocalcin release was determined in cell supernatants with an enzyme immunoassay as described previously [23,34]. Collagen synthesis was determined as the *de novo *release of the carboxy-terminal peptide fragment of collagen type I (CICP), reflecting true collagen synthesis. CICP was determined with a selective ELISA (Quidel Corporation, Cedarlane, Hornby, Ontario, Canada) in conditioned medium from confluent normal and OA osteoblasts incubated for 48 hours in Ham's F12/DMEM medium containing 0.5% BSA. CICP release was then reported as ng per mg of cellular protein.

### Statistical analysis

All quantitative data are expressed as means ± SEM. The data were analyzed with Student's *t *test; and *p *< 0.05 was considered statistically significant.

## Results

### Determination of two population of OA patients

**Figure 1:**
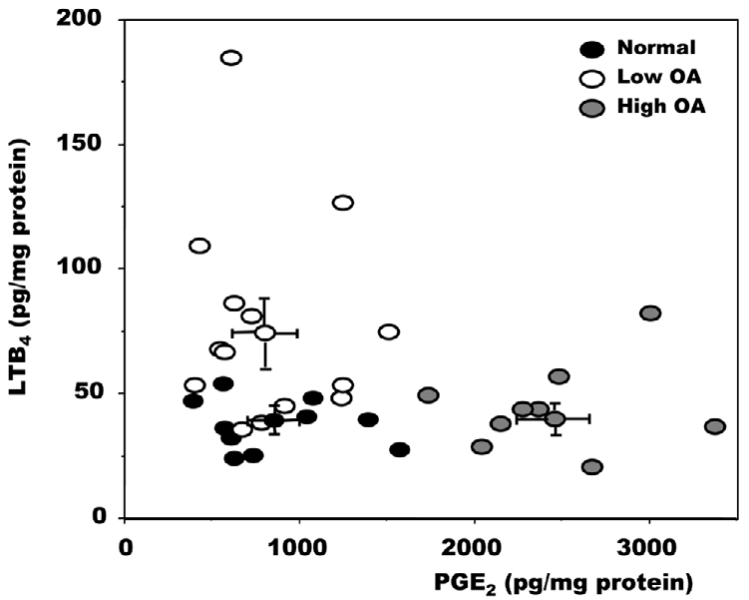
Relationship between PGE_2 _and LTB_4 _levels in normal and osteoarthritis osteoblasts. Cells were grown to confluence in Ham's F12/DMEM medium containing 10% fetal bovine serum. Confluent cells were incubated for their last 48 hours in serum-free medium containing 1% insulin-transferrin-selenium mix (ITS). Prostaglandin E_2 _(PGE_2_) and leukotriene B_4 _(LTB_4_) levels were measured with selective ELISA. Data points represent values for individual cell cultures. Low osteoarthritis (OA) and high OA were separated on the basis of their PGE_2 _levels: high OA had PGE_2 _levels at least 2 SD above the mean normal PGE_2 _levels. Normal samples, *n *= 10; low OA, *n *= 14; high OA, *n *= 8.

As we observed previously [23,34], OA osteoblasts presented an altered phenotype from that of normal osteoblasts. This was demonstrated by an elevated alkaline phosphatase activity and osteocalcin release in response to stimulation with 1,25(OH)_2_D_3_, and an enhanced production of collagen type I (Table 1). Under the present culture conditions, osteoblasts expressed bone-specific type I collagen without any contamination from cartilage-specific type II collagen [23]. Osteoblasts isolated from OA patients also presented variable endogenous PGE_2 _production, as shown previously [12,23]. We therefore separated our OA patients into low and high categories on the basis of PGE_2 _production, with reciprocal levels of LTB_4 _produced by these cells (Figure 1). However, regardless of their endogenous production of PGE_2_, phenotypic characteristics were similar between cell cultures of OA osteoblasts as previously reported [23], and this variable PGE_2 _production was due to alterations in COX-2 production [43].

### Regulation of expression of 5-LO and FLAP

**Figure 2:**
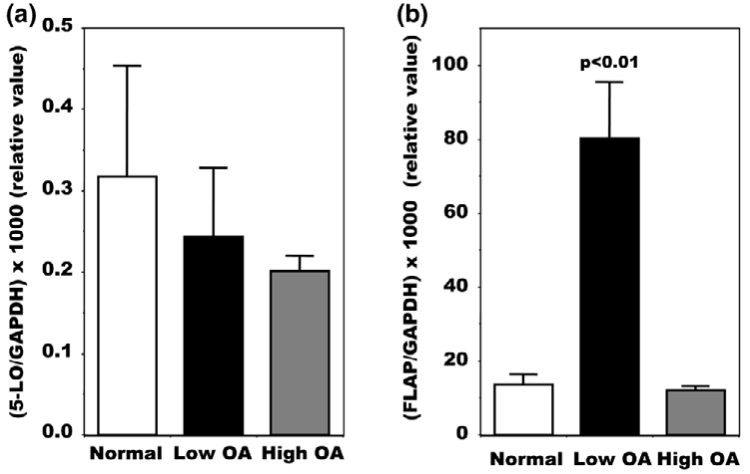
Real-time RT-PCR analysis of 5-LO and FLAP mRNA isolated from normal and osteoarthritis osteoblasts. Confluent cells were incubated for their last 48 hours in Ham's F12/DMEM medium containing 0.5% BSA. Cells were lyzed with TRIzol and RNA extracted according to the protocol described in the Materials and methods section. Plasmid DNAs containing the target gene sequences were used to generate standard curves. When comparing normal and osteoarthritis (OA) osteoblast expression levels, values were converted to numbers of molecules and the values for each sample were calculated as the ratio of the number of molecules of 5-lipoxygenase (5-LO) **(a) **or 5-LO-activating protein (FLAP) **(b) **to the number of molecules of glyceraldehyde-3-phosphate dehydrogenase (GAPDH). Values are means ± SEM for *n *= 4 samples for all conditions.

**Figure 3:**
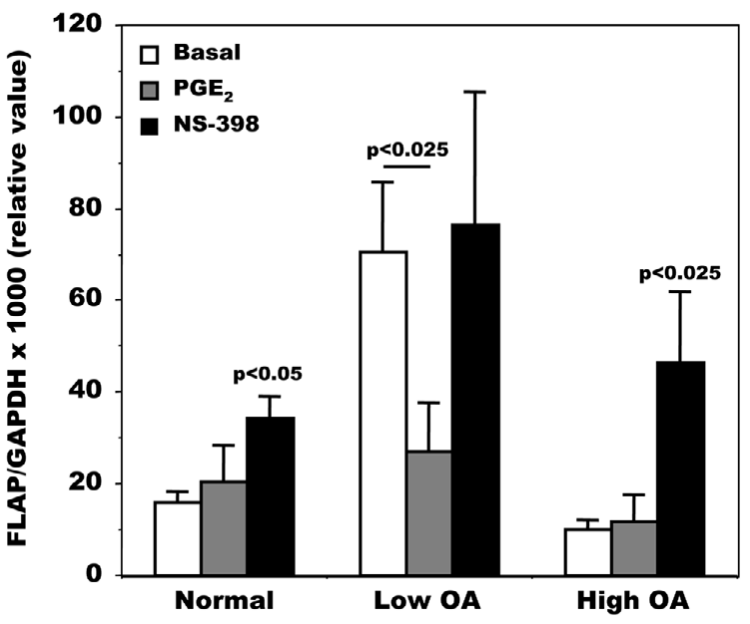
Regulation of FLAP mRNA expression by PGE_2 _and NS-398 in normal and osteoarthritis osteoblasts. Confluent cells were incubated for their last 48 hours in Ham's F12/DMEM medium containing 0.5% BSA in the presence or absence of 500 nM prostaglandin E_2 _(PGE_2_) or 10 μM NS-398. 5-Lipoxygenase-activating protein (FLAP) expression was measured as described in the legend to Figure 2. Values are means ± SEM; *p *values compare data with basal values in each group. Normal samples, *n *= 3; low and high osteoarthritis (OA), *n *= 5 for each group. GAPDH, glyceraldehyde-3-phosphate dehydrogenase.

On the basis of PGE_2 _production, we next questioned what mechanism or mechanisms were responsible for the alteration of LTB_4 _production. We measured the expression of the two key enzymes involved in LT production, 5-LO and FLAP (Figure 2). The expression of 5-LO, although slightly lower in the high OA group, was not significantly different between normal and OA patients (Figure 2a), yet the expression of FLAP was variable (Figure 2b). Indeed, FLAP expression was highest in those patients with the lowest PGE_2 _levels, whereas FLAP expression was similar to normal in OA osteoblasts with the highest PGE_2 _levels. To determine the mechanism responsible for this shunt between the production of PGE_2 _and that of LTB_4 _in OA osteoblasts, we then determined the effect of the addition of PGE_2 _to osteoblasts or of an inhibitor of PGE_2 _production on the expression of FLAP by real-time PCR. As shown in Figure 3, FLAP expression in normal cells did not vary much with the applied treatments except for a small but significant increase in response to NS-398, a selective COX-2 inhibitor. In low OA osteoblasts, PGE_2 _treatments decreased FLAP expression about 2.5-fold (*p *< 0.025), whereas inhibiting endogenous PGE_2 _production with NS-398 did not inhibit FLAP expression. In high OA osteoblasts with already high PGE_2 _levels, the addition of PGE_2 _failed to modify FLAP expression, whereas inhibiting PGE_2 _production with NS-398 enhanced FLAP expression about 4-fold (*p *< 0.025). Such an increase in FLAP expression in response to NS-398 could explain our previous observation of an increase in LTB_4 _production by OA osteoblasts under similar conditions [12]. In contrast, under similar experimental conditions with PGE_2 _or NS-398, 5-LO expression did not vary significantly in either normal or OA osteoblasts (not shown).

**Figure 4:**
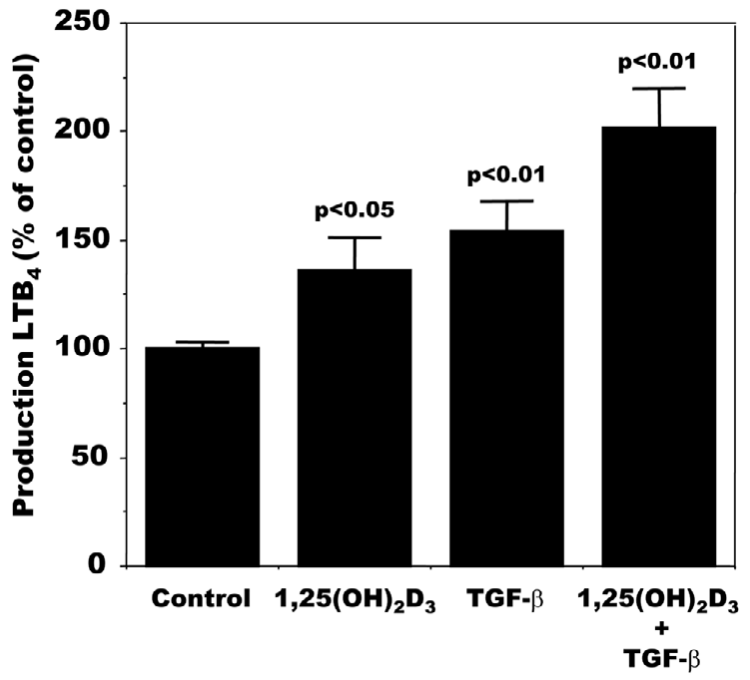
Modulation of LTB_4 _production by 1,25(OH)_2_D_3 _and TGF-β in osteoarthritis osteoblasts. Confluent cells were incubated for their last 48 hours in Ham's F12/DMEM medium containing 2% fetal bovine serum in the presence or absence of 50 nM 1,25-dihydroxyvitamin D_3 _(1,25(OH)_2_D_3_), 10 ng/ml transforming growth factor-β (TGF-β), or both. Leukotriene B_4 _(LTB_4_) was measured in conditioned medium with the use of a very selective ELISA. Values are means ± SEM for *n *= 4 samples for all groups.

**Figure 5:**
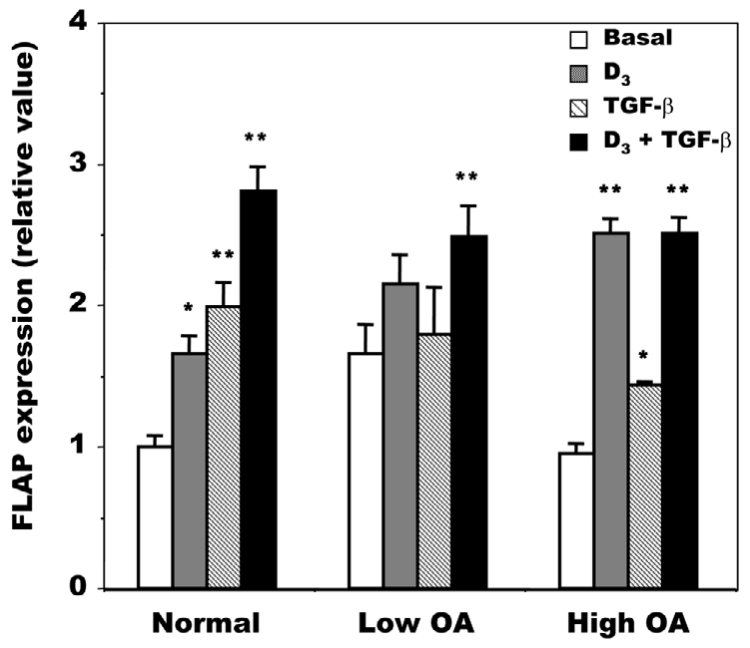
Regulation of FLAP mRNA expression by 1,25(OH)_2_D_3 _and TGF-β in normal and osteoarthritis osteoblasts. Confluent cells were incubated for their last 48 hours in Ham's F12/DMEM medium containing 0.5% BSA in the presence or absence of 50 nM 1,25-dihydroxyvitamin D_3 _(1,25(OH)_2_D_3_; D_3_), 10 ng/ml transforming growth factor-β (TGF-β), or both. Osteoarthritis (OA) osteoblasts were separated into low and high OA as described in the legend to Figure 1. 5-Lipoxygenase-activating protein (FLAP) expression was measured as described in the legend to Figure 2. Values are means ± SEM. Normal samples, *n *= 3; low and high OA, *n *= 4 for each group.

We then evaluated the modulation of LTB_4 _production in OA osteoblasts by 1,25(OH)_2_D_3_, transforming growth factor-β (TGF-β) or a combination of the two because both factors have been shown to modulate the activity and/or expression of FLAP in other cell systems [28,30,31,44-46]. As shown in Figure 4, OA osteoblasts responded to 1,25(OH)_2_D_3_, TGF-β or a combination of the two with an increase in LTB_4 _production, regardless of their endogenous PGE_2 _production; data were therefore pooled for both groups of OA osteoblasts. The effect of 1,25(OH)_2_D_3 _and TGF-β was additive in this particular setting. This effect was not due to any significant modification of 5-LO expression in these cells (not shown). In contrast, when we evaluated the expression of FLAP, this varied with the applied treatment (Figure 5). In addition, the expression of FLAP was variable in response to TGF-β treatment depending on the subgroup (low or high) of OA patients.

**Figure 6:**
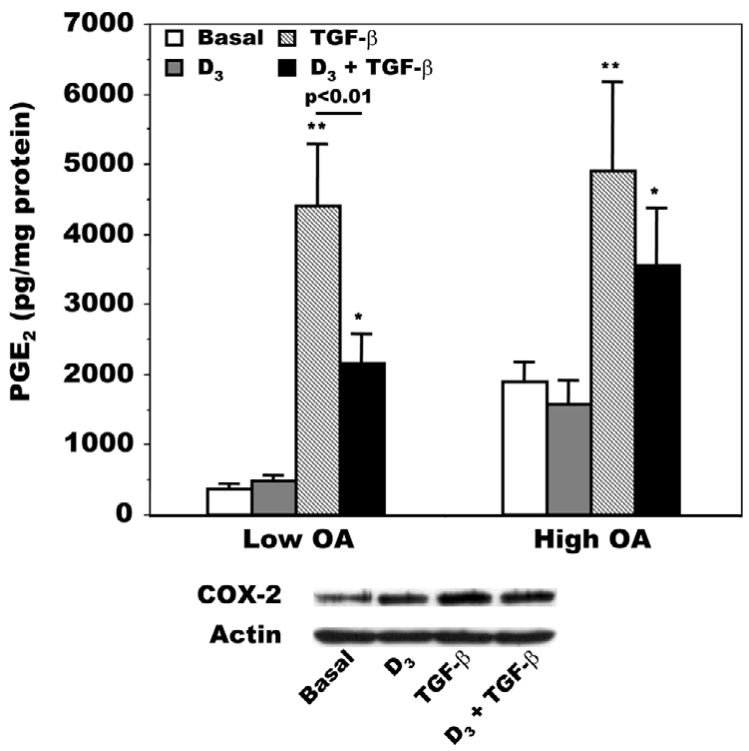
Modulation of PGE_2 _production by 1,25(OH)_2_D_3 _and TGF-β in osteoarthritis osteoblasts. Confluent cells were incubated for their last 48 hours in Ham's F12/DMEM medium containing 0.5% BSA in the presence or absence of 50 nM 1,25-dihydroxyvitamin D_3 _1,25(OH)_2_D_3_; D_3_), 10 ng/ml transforming growth factor-β (TGF-β), or both. Top: prostaglandin E_2 _(PGE_2_) was measured in conditioned medium with the use of a very selective ELISA. Values are means ± SEM for *n *= 7 preparations for both low and high osteoarthritis (OA) osteoblast groups. *p < 0.01; **p < 0.05 compared with the respective basal value for the low or high OA group. Bottom: representative Western blot analysis of cyclooxygenase-2 (COX-2) production in five OA osteoblast preparations in response to 1,25(OH)_2_D_3_, TGF-β, or both. Loading between samples was measured by western blot analysis of actin. Low OA and high OA, *n *= 7 for each group for PGE_2 _determinations.

Because we observed variations in LTB_4 _production (Figure 4) and FLAP expression (Figure 5) in response to 1,25(OH)_2_D_3_, TGF-β or both, we next examined whether PGE_2 _production varied with these treatments and whether this could be linked with a modulation of COX-2 synthesis. PGE_2 _production by isolated OA osteoblasts was enhanced by TGF-β in both the low and high OA subgroups of patients (Figure 6), whereas 1,25(OH)_2_D_3 _was without significant effect in both groups. However, whereas 1,25(OH)_2_D_3 _significantly inhibited the stimulating effect of TGF-β in the low OA subgroup, it was without significant effect in the high OA subgroup (Figure 6). This effect of TGF-β and 1,25(OH)_2_D_3 _on PGE_2 _production was reflected by a similar effect on COX-2 synthesis responsible for PGE_2 _production (Figure 6, bottom panel). Indeed, TGF-β significantly increased COX-2 synthesis by about 97 ± 37% (*p *< 0.05 versus basal values), and the addition of 1,25(OH)_2_D_3 _with TGF-β caused a small decrease compared with TGF-β alone (42 ± 17% decrease, *p *< 0.05).

### Relationship between PGE_2 _levels and IL-10 and IL-18

**Figure 7:**
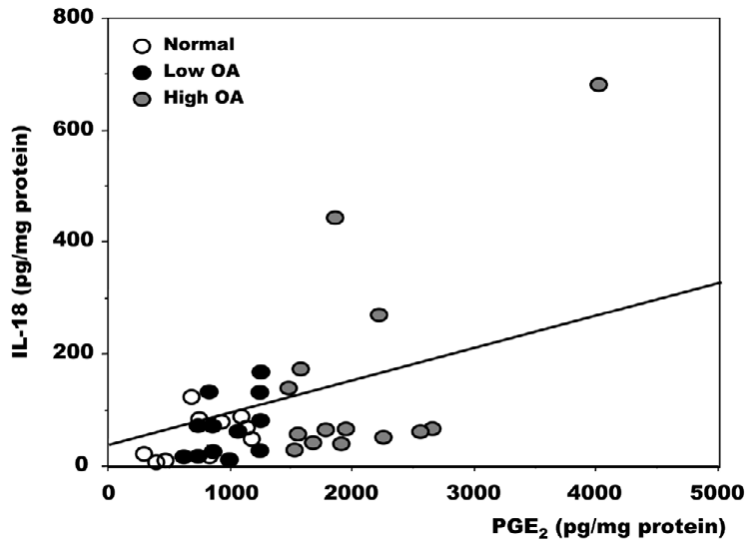
Relationship between PGE_2 _and IL-18 levels in normal and osteoarthritis osteoblasts. Confluent cells were incubated for their last 48 hours in serum-free medium containing 1% insulin-transferrin-selenium mix (ITS). Prostaglandin E_2 _(PGE_2_) and IL-18 levels were measured in supernatants with the use of selective ELISA. Data are values for individual cell cultures. Osteoarthritis (OA) osteoblasts were separated into low and high OA as described in the legend to Figure 1. Normal samples, *n *= 10; low OA, *n *= 12; high OA, *n *= 14 samples.

In macrophages, the shunt from the COX to the 5-LO pathway is linked with a variable production of IL-10; as PGE_2 _levels rise, IL-10 levels increase and inhibit the synthesis of LTB_4 _[24]. Unfortunately, in our cell culture system IL-10 levels were very low, close to the detection limit, and failed to vary with PGE_2 _levels (not shown). A link between IL-18 and PGE_2 _levels in the synovial fluid of patients with OA of the knee has also been established [47], and IL-18 upregulates LTs production in neutrophils [32]. In OA osteoblasts, the levels of IL-18 were generally slightly higher than normal. However, no clear relationship between IL-18 and PGE_2 _levels could be observed in OA osteoblasts except in a limited subgroup of patients (Figure 7).

## Discussion

This study provides the first comprehensive explanation about the regulation of the expression of the enzymatic system responsible for LT synthesis in osteoblasts. It also revealed new and unique mechanisms of regulation of FLAP expression in OA osteoblasts. This is of special interest because the exact mechanism underlying a shunt from the COX to the 5-LO pathway in osteoblasts remains unknown. However, this knowledge could be crucial for therapeutic intervention in OA. The actual therapies for OA are somewhat limited to a decrease in pain in affected joints with the use of either non-selective NSAIDs [48-50] or the selective coxibs [48,51-53]. These later compounds are aimed at decreasing the inducible COX-2 activity responsible for prostaglandin synthesis. The long-term treatment of OA patients with selective inhibitors of COX-2 could promote the production of LTs. In an animal model of arachidonic acid-induced inflammation, Goulet and colleagues [54] showed that an NSAID can suppress almost completely the inflammatory response in 5-LO-deficient mice, in contrast with wild-type animals. Hence, PGE_2 _produced via both COX-1 and COX-2 could modulate the activity of 5-LO in these animals, which is reminiscent of the fact that PGE_2 _inhibits 5-LO activity [55].

We showed previously that in OA subchondral osteoblasts long-term treatment with NS-398, a selective COX-2 inhibitor, leads to an increase in LTB_4 _production [12]. The present study demonstrated that the induction of LTB_4 _production in response to NSAIDs or NS-398 in OA osteoblasts involved no major modification of 5-LO expression, in contrast to the situation observed in OA chondrocytes [56]. In fact, the increase in LTB_4 _was either due to an upregulation of FLAP expression in high OA osteoblasts in response to inhibitors of PGE_2 _production or it was already high in the low OA subgroup. FLAP is known to present arachidonic acid to 5-LO for its synthesis into LTs [25,27]. Furthermore, this elevated FLAP expression could be curbed by exogenous PGE_2_. This was especially true in the low OA subgroup presenting low PGE_2 _levels, whereas in the high OA subgroup with already high PGE_2 _levels the addition of exogenous PGE_2 _failed to modify FLAP expression. These results indicate that the balance of PGE_2 _levels in OA osteoblasts is actually driving the expression of LTs, and conversely that chronic inhibition of COX may be leading to the synthesis of LTs. Given that LTs are more pro-inflammatory than prostaglandins, this would suggest that chronic inhibition of COX could lead to potential harmful effects. Such a shunt from the COX to the 5-LO pathway has previously been observed in other cell systems, in particular in macrophages [13,14,57].

This shunt was either related to an increase in 5-LO expression or that of FLAP through an IL-10-dependent or IL-18-dependent pathway in other cell systems. Indeed, PGE_2_-driven IL-10 synthesis in monocytes/macrophages *in vitro *has been shown to inhibit LTB_4 _production by these cells [24], whereas in neutrophils IL-18 stimulates the synthesis of LTs [32]. In subchondral osteoblasts, a link between IL-10 and PGE_2 _could not be established because IL-10 synthesis by either normal or OA osteoblasts was very low, close to the limit of detection. This possibility therefore seems very weak. In addition, endogenous IL-18 levels in subchondral osteoblasts were also weakly linked with endogenous PGE_2 _levels. In the synovial fluid of patients with OA of the knee, a linear relationship has been established between IL-18 and PGE_2 _or IL-6 [47]. Indeed, in OA osteoblasts treated with either an inhibitor of PGE_2 _synthesis or exogenous PGE_2 _we observed a slight decrease in IL-18 or a slight increase, respectively (not shown). However, considering the relationship established between PGE_2 _and IL-18 in this study it would be surprising if IL-18 were to have a significant role.

The modulation of LTB_4 _production in OA osteoblasts was also linked to alterations of FLAP expression in these cells in response to 1,25(OH)_2_D_3 _and TGF-β as observed in other cell systems [30,31,44,58]. However, this regulation by 1,25(OH)_2_D_3 _and TGF-β seemed to be linked with the actual physiological state of the cells. Indeed, low PGE_2 _OA osteoblast producers responded more readily than normal osteoblasts to stimulation with 1,25(OH)_2_D_3_. In contrast, the response to TGF-β challenge was somewhat offset in both low and high PGE_2 _producers. This might have been due to the endogenous high TGF-β levels produced by all OA osteoblasts [23] and hence to a possible chronic desensitization to further TGF-β challenge *in vitro*. However, concomitant incubation with 1,25(OH)_2_D_3 _and TGF-β was able to stimulate FLAP expression to the levels observed in normal cells under similar conditions. Again, this would suggest that FLAP is the key enzyme that controls the production of LTs in OA osteoblasts, rather than 5-LO, which showed little variation of expression regardless of treatment. This is in contrast to the situation observed with several cell systems in which 1,25(OH)_2_D_3 _and TGF-β enhanced the expression of 5-LO [28,29,45,46] or both 5-LO and FLAP [44]. Because the activity of 5-LO can also be modulated by its phosphorylation state [59], this could also be a possible mechanism of control in OA osteoblasts; this was not investigated in this study.

However, the effect of 1,25(OH)_2_D_3 _and TGF-β on PGE_2 _production is different from that on LTB_4 _production in OA osteoblasts. Indeed, TGF-β stimulated PGE_2 _production to similar levels in both the low and high OA subgroups, a situation linked with its modulation of COX-2 synthesis. In contrast, the effect of 1,25(OH)_2_D_3 _on PGE_2 _production was weaker than that on LTB_4 _production, also reflected by its effect on COX-2 synthesis. On its own 1,25(OH)_2_D_3 _had no effect, whereas it inhibited the effect of TGF-β in the low OA osteoblasts subgroup only. In normal human osteoblasts, 1,25(OH)_2_D_3 _was previously shown to inhibit PGE_2 _production both alone and in response to TGF-β [60], a situation similar to our low OA osteoblasts subgroup. In contrast, in the mouse osteoblast-like MC3T3-E1 cells, 1,25(OH)_2_D_3 _is without effect, whereas it inhibits PGF-2α-induced PGE_2 _production [61]. TGF-β alone can stimulate PGE_2 _production in serum-free conditions in MC-3T3-E1 cells and can potentiate the effect of IL-1, a situation not observed in the presence of 10% serum [62]. As our assays were performed in serum-free conditions for PGE_2 _production, our data are similar to those in this situation. Taken together, the results for LTB_4 _and PGE_2 _production in response to 1,25(OH)_2_D_3 _and TGF-β indicate that the production of LTB_4 _is more sensitive to 1,25(OH)_2_D_3 _treatment through its effect on FLAP expression, especially in the high OA osteoblasts group, whereas the production of PGE_2 _is sensitive to TGF-β in both groups. Moreover, the overall effect of 1,25(OH)_2_D_3 _and TGF-β would promote both PGE_2 _and LTB_4 _production in all OA osteoblasts, whereas their effect is more evident on the production of PGE_2_.

Although OA osteoblasts could separate OA patients into two groups producing either low or high levels of PGE_2_, these cells showed similar phenotypic characteristics and produced similar levels of collagen type 1, although at higher levels than in normal osteoblasts. This suggests that neither PGE_2 _nor LTB_4 _has a direct role on bone tissue sclerosis in OA. However, elevated LTB_4 _levels could locally influence bone resorption, leading to an increase in bone resorption indices. Clinical studies have reported both increases and an absence of change in bone resorption parameters in OA patients [63-70], a situation that could be linked with the endogenous PGE_2 _production by OA bone tissue and thereby that of LTB_4_. Indeed, some authors have suggested that patients with progressive knee OA had increased bone resorption parameters. Last, osteoblasts were prepared from the overall subchondral bone plate of the tibial plateaus of OA patients, thus not isolating subchondral bone from lesional and non-lesional areas of articular cartilage. Although this may be considered a limitation of the present study, our own previous results by using osteoblasts isolated from bone tissue underlying lesional and non-lesional areas of cartilage did not show any overt differences in terms of phenotype or behavior [71]. Moreover, OA osteoblasts isolated from the trabecular bone region below the subchondral bone plate show similar results to those of OA osteoblasts from the subchondral bone plate [72,73], and OA bone tissue from non-weight-bearing areas also show similar alterations to those in joints [74,75], thus indicating that the bone tissue alterations in OA patients are generalized rather than localized events.

## Conclusion

We have shown that in OA osteoblasts the synthesis of LTs is linked to the tight regulation of FLAP expression, not that of 5-LO. Moreover, the basal synthesis of LTs is linked to a variable expression of FLAP in OA osteoblasts as a result of their endogenous production of PGE_2_. Both 1,25(OH)_2_D_3 _and TGF-β modulated the expression of FLAP and thereby that of LTB_4_. IL-10 is not involved in the regulation of the synthesis of LTs in OA osteoblasts, whereas IL-18 levels are linked with PGE_2 _levels.

## Abbreviations

1,25(OH)_2_D_3 _= 1,25-dihydroxyvitamin D_3_; 5-LO = 5-lipoxygenase; BSA = bovine serum albumin; CICP = carboxy-terminal peptide fragment of collagen type I; COX-2 = cyclooxygenase-2; ELISA = enzyme-linked immunosorbent assay; FLAP = 5-lipoxygenase-activating protein; GAPDH = glyceraldehyde-3-phosphate dehydrogenase; IL = interleukin; LTB_4 _= leukotriene B_4_; LTs = leukotrienes; NSAIDs = non-steroidal anti-inflammatory drugs; OA = osteoarthritis; PGE_2 _= prostaglandin E_2_; RT-PCR = reverse transcriptase-mediated polymerase chain reaction; TGF-β = transforming growth factor-β.

## Competing interests

The authors declare that they have no competing interests.

## Authors' contributions

KM performed most of the experiments and wrote the first draft of the manuscript. AD performed cell cultures and some experiments. JM-P and J-PP were responsible for manuscript writing and discussion of results. ND provided OA knee samples and contributed to the discussion. DL proposed the original concepts, planned and performed some experiments, participated in the discussion and wrote the final version of the manuscript. All authors read and approved the final manuscript.

## Figures and Tables

**Table 1:** Evaluation of phenotypic markers of normal and osteoarthritis (OA) subchondral osteoblasts

Source	Alkaline phosphatase (nmol/mg protein/30 min)	Osteocalcin (ng/mg protein/48 hours)	Collagen type I (ng/mg protein/48 hours)
Normal (*n *= 11)	624.7 ± 88.8	176.6 ± 24.7	285.3 ± 17.1
OA (*n *= 26)	1333.1 ± 215.7	264.0 ± 20.7	370.4 ± 8.1
	*p *< 0.005	*p *< 0.05	*p *< 0.015

Confluent osteoblasts were incubated for their last 2 days of culture in Ham's F12/DMEM medium containing 2% charcoal-treated fetal bovine serum and 50 nM 1,25-dihydroxyvitamin D_3_. Values are means ± SEM. The statistical analysis compared OA values with their respective normal values.
